# Pyometra by Clostridium clostridioforme: A Case Report

**DOI:** 10.7759/cureus.93108

**Published:** 2025-09-24

**Authors:** Minoru Sakakiyama, Koji Hayashi, Maho Hayashi, Yuzuru Takeuchi

**Affiliations:** 1 Department of Internal Medicine, Fukui General Hospital, Fukui, JPN; 2 Department of Endocrinology and Metabolism, University of Fukui, Fukui, JPN; 3 Department of Rehabilitation Medicine, Fukui General Hospital, Fukui, JPN; 4 Department of Obstetrics and Gynecology, Fukui General Hospital, Fukui, JPN

**Keywords:** clostridium, clostridium clostridioforme, enterococcus faecalis, proteus mirabilis, pyometra

## Abstract

We report a rare case of pyometra caused by *Clostridium clostridioforme* (*C. clostridioforme*) in an elderly woman, illustrating the challenges of managing uncommon microbial etiologies in uterine infections. A 91-year-old woman with a history of paroxysmal atrial fibrillation, type 2 diabetes, and neurogenic bladder developed persistent high fever (>38.5°C) over five days, despite prior antibiotic therapy with ceftriaxone for suspected urinary tract infection. Imaging revealed fluid accumulation within the uterine cavity, consistent with pyometra. Initial treatment with meropenem (MEPM) rapidly improved her condition, with fever and CRP levels decreasing. Cultures from uterine lavage identified *Proteus mirabilis* and *Enterococcus faecalis*, both susceptible to MEPM, and her symptoms temporarily resolved, leading to discontinuation of antibiotics.

However, five days post-discharge, she experienced recurrent high fever, and repeat lavage revealed *C. clostridioforme* as the causative pathogen, confirmed by culture and susceptibility testing. Despite oral antibiotics with faropenem and later metronidazole, her fever persisted, necessitating readmission. Daily uterine lavages and intermittent intravaginal metronidazole suppositories gradually led to clinical improvement; the purulent discharge diminished, and body temperature normalized. The infection finally resolved after ongoing local management and a short course of antibiotics for a subsequent urinary tract infection.

This case highlights the importance of considering atypical pathogens such as *C. clostridioforme* in refractory pyometra, especially in elderly or immunocompromised patients. It underscores the need for combined systemic and local therapies and the significance of microbial susceptibility testing to guide treatment. The report emphasizes that managing complex uterine infections requires an integrated approach, and further research is necessary to understand the pathophysiology and optimal management of *C. clostridioforme*-related pyometra.

## Introduction

*Clostridium clostridioforme* (*C. clostridioforme*), recently reclassified as *Enterocloster clostridioforme*, is characterized as anaerobic, fusiform rods [1]. It typically stains Gram-negative, despite belonging to a genus often associated with Gram-positive bacteria, and its bacilli are frequently observed in pairs with tapered ends [2]. Spores are difficult to find, and when present, the cells do not show oddly shaped or swollen forms [1,2]. It is nonproteolytic and utilizes simple carbohydrates for growth [1].

*C. clostridioforme* is commonly human gut associated and is a prevailing member of the normal intestinal microbiota [1,3]. While initially isolated from calf rumen, it has more recently and frequently been found in various human clinical specimens [1,2]. It can also be found in other body sites, such as subgingival microflora in AIDS patients and vaginal flora in women [2]. This bacterium is recognized as an opportunistic pathogen and has been implicated in a variety of serious infections, particularly when the natural intestinal barrier is compromised [1-3]. It is associated with more serious or invasive human infections compared to *Clostridium bolteae* and *Clostridium hathewayi* [2]. Documented infections include bacteremia (often found in pure culture and sometimes associated with septic shock), intra-abdominal and pelvic abscesses (commonly linked to perforated viscera, appendicitis, or pancreatitis), peritonitis and wound infections (including postoperative), ventriculoperitoneal shunt infections, paravertebral and retrorectal abscesses, meningitis, chronic osteomyelitis, and pleural empyema [2]. Therefore, *C. clostridioforme* is clinically significant due to its potential to cause severe and sometimes life-threatening infections.

In terms of antimicrobial susceptibility, *C. clostridioforme* commonly produces β-lactamase and exhibits resistance to penicillin G, whereas it is generally susceptible to metronidazole (MNZ), ampicillin/sulbactam, and piperacillin/tazobactam [2]. It should also be noted that VanB-type vancomycin resistance has been reported in *C. clostridioforme* [4]. Therefore, the observed resistance patterns raise the possibility of therapeutic difficulties, with a consequent increase in clinical burden, including longer durations of therapy, use of non-standard or combination regimens, and heightened monitoring for adverse events.

Notably, *C. clostridioforme* has rarely been reported as a causative agent of gynecological infections. Here, we present a rare case of pyometra caused by *C. clostridioforme*, which emphasizes the potential significance of this organism in female reproductive tract infections and highlights the need for awareness among clinicians.

## Case presentation

**Figure 1:**
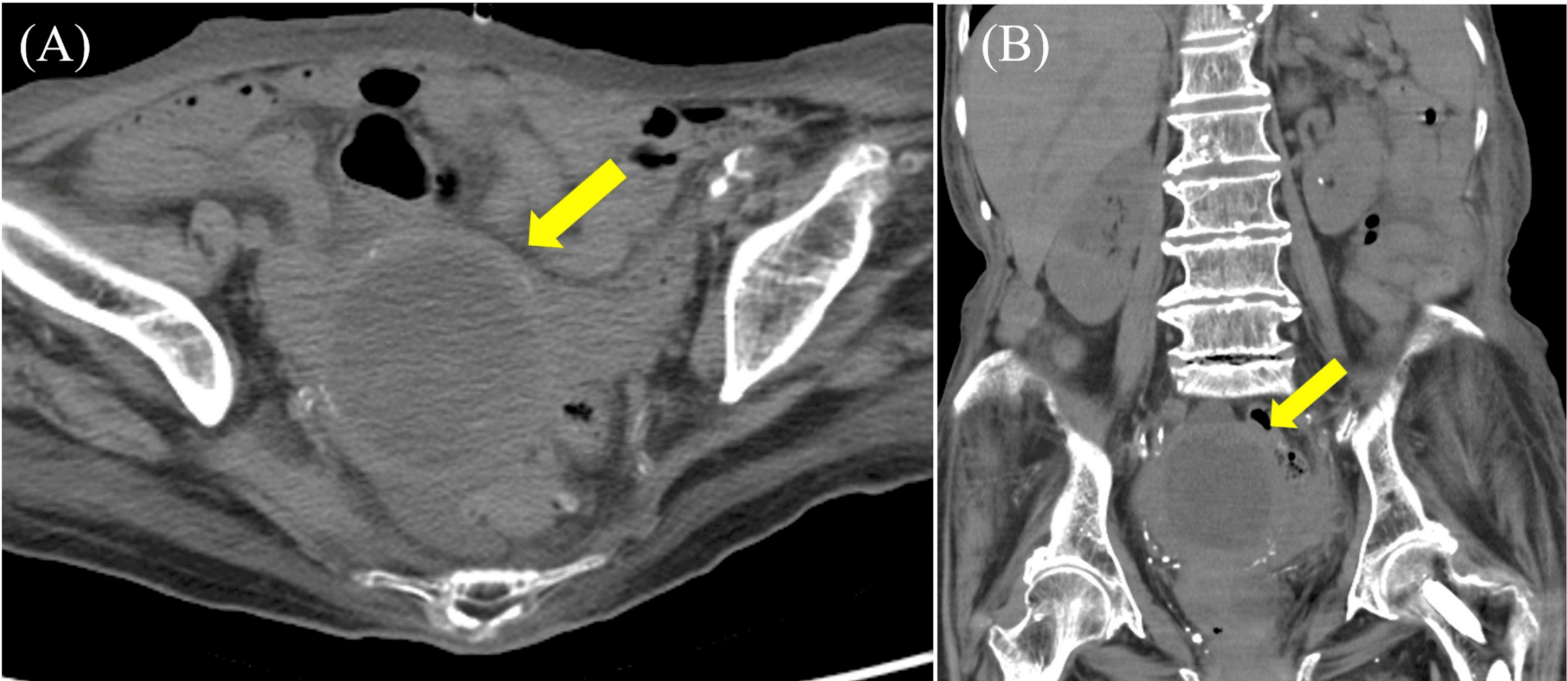
Pelvic computed tomography (CT) findings. Pelvic CT demonstrating a distended uterine cavity filled with fluid or purulent material, appearing as a well-defined, fluid-density structure within the uterus. (A) Axial section; (B) coronal section.

A 91-year-old woman with a history of paroxysmal atrial fibrillation, type 2 diabetes mellitus, and neurogenic bladder managed with medication developed a high fever exceeding 38.5°C over five days. Although previously hospitalized and treated with ceftriaxone 2 g/day for a suspected urinary tract infection, she was referred to our hospital due to persistent high fever (around 40°C). Upon admission, laboratory findings included leukocytosis with neutrophilia and an elevated C-reactive protein (CRP) level of 16.83 mg/dL (Table 1), despite the absence of pyuria. Pelvic computed tomography (CT) revealed fluid retention within the uterine cavity, suggestive of pyometra (Figure 1). Meropenem (MEPM) 1 g/day was initiated.

**Table 1:** The blood test results.

Inspection items	Result	Reference range
Red blood cell	337 × 10⁴/μL	435-555 × 10⁴
White blood cell	20100/μL	3300-8600
Hemoglobin	9.7 g/dL	13.7-16.8
Platelet	63 × 10⁴/μL	15.8-34.8 × 10⁴
Blood glucose	223 mg/dL	73-109
Glycated hemoglobin (HbA1c)	6.7%	4.9-6.0
Total protein	5.6 g/dL	6.6-8.1
Uric acid	0.62 mg/dL	2.6-7.0
Total bilirubin	0.5 mg/dL	0.4-1.2
Aspartate aminotransferase	23 U/L	13-30
Alanine aminotransferase	14 U/L	7-30
Alkaline phosphatase	112 U/L	38-113
Lactate dehydrogenase	170 U/L	124-222
γ-Glutamyltransferase	12 U/L	9-32
Creatine phosphokinase	10 U/L	41-153
Cholinesterase	171 U/L	201-421
Amylase	30 U/L	44-132
Blood urea nitrogen	18.1 mg/dL	8.0-20.0
Creatinine	0.62 mg/dL	0.65-1.07
Sodium	132 mmol/L	138-145
Potassium	4.3 mmol/L	3.6-4.8
Chloride	94 mmol/L	101.0-108.0
eGFR	66.3 mL/minute/1.73m²	≥ 60
C-reactive protein (CRP)	16.83 mg/dL	0.0-0.14

On day 2, uterine lavage revealed approximately 140 mL of grayish purulent fluid. Cultures grew* Proteus mirabilis* (*P. mirabilis*) and *Enterococcus faecalis* (*E. faecalis*), both susceptible to MEPM in vitro (Tables 2-3). On day 8, as her high fever resolved and CRP decreased to 1.25 mg/dL, MEPM was discontinued. On day 9, repeat uterine lavage drained approximately 70 mL of purulent fluid. Cytological examination of the endometrium showed no malignant cells, and contrast-enhanced pelvic magnetic resonance imaging (MRI) did not reveal any malignant lesions. Although low-grade fever around 37°C persisted, her condition stabilized, and she was discharged on day 11.

**Table 2:** Antimicrobial susceptibility testing results for Proteus mirabilis. R, resistant; S, sensitive

Antibiotic	Minimum inhibitory concentration	Sensitivity
Ampicillin	<8	S
Cefazolin	<8	S
Cefotiam	<8	S
Cefmetazole	<16	S
Cefepime	<8	S
Flomoxef	<8	S
Meropenem	<1	S
Gentamicin	<4	S
Minocycline	>8	R
Ciprofloxacin	<1	S
Levofloxacin	<2	S
Fosfomycin	<4	S
Trimethoprim-sulfamethoxazole	<0.5	S

**Table 3:** Antimicrobial susceptibility testing results for Enterococcus faecalis. NA, not available for interpretation; R, resistant; S, sensitive

Antibiotic	Minimum inhibitory concentration	Sensitivity
Ampicillin	2	S
Cefazolin	16	NA
Cefotiam	>16	NA
Cefmetazole	>32	NA
Cefepime	>16	NA
Flomoxef	>16	NA
Meropenem	<4	S
Gentamicin	8	NA
Minocycline	>8	R
Levofloxacin	<1	S
Fosfomycin	>16	NA
Vancomycin	<2	S
Trimethoprim-sulfamethoxazole	<2	NA

After discharge, she developed a high fever ranging from 38°C to 39°C and visited our hospital on day 5 post-discharge. Repeat uterine lavage drained approximately 150 mL of pus, and culture from the lavage later grew *C. clostridioforme* (antibacterial susceptibility is shown in Table 4). Outpatient follow-up was planned, and she was prescribed oral faropenem, which was later switched to MNZ (1500 mg/day) due to insufficient clinical improvement. Despite the switch to MNZ, her fever persisted, and she was readmitted on day 11 post-discharge. After readmission, she underwent daily uterine lavage. By day 5 post-readmission, the volume of purulent discharge had decreased to 15 mL, and her temperature normalized to 36°C. Consequently, MNZ was discontinued. From day 9 post-readmission, drainage diminished to less than 10 mL. Uterine lavage was maintained two to three times per week, with intravaginal chloramphenicol suppositories (100 mg) administered immediately after the uterine lavage.

**Table 4:** Antimicrobial susceptibility testing results for Clostridium clostridioforme. The minimum inhibitory concentration (MIC) values for *Clostridioides clostridioforme* were not available. R, resistant; S, sensitive

Antibiotic	Sensitivity
Ampicillin-sulbactam	R
Cefazolin	R
Cefotiam	R
Cefepime	R
Flomoxef	R
Meropenem	S
Gentamicin	R
Minocycline	R
Clindamycin	S
Levofloxacin	R
Fosfomycin	R
Vancomycin	S
Trimethoprim-sulfamethoxazole	R

On day 22 post-readmission, she developed signs suggestive of urinary tract infection (UTI), for which she received a five-day course of oral amoxicillin/clavulanate. By day 26 post-readmission, the purulent discharge had completely ceased. The patient remained afebrile and was discharged back to her nursing facility on day 33 post-readmission. Because she was discharged to a nursing facility, further follow-up was not conducted at our institution.

## Discussion

This report describes a rare case of pyometra caused by *C. clostridioforme*. Notably,* P. mirabilis* and* E. faecalis* were initially isolated from uterine lavage cultures, with *C. clostridioforme* subsequently detected during the course of treatment. To our knowledge, no reports have described changes in the bacterial flora of pyometra during treatment or C. clostridioforme as the causative agent.

*Clostridium *species are well recognized as important pathogens in gynecology, particularly as causative agents of severe postoperative infections in obstetric and gynecologic settings. *Clostridium* species can be isolated from the genital tract of approximately 10% of women as part of the normal vaginal microbiota [5]. Although uncommon, infections due to *Clostridium perfringens* (*C. perfringens*) or *Clostridium sordellii* (*C. sordellii*) after childbirth or abortion can be severe [6]. Uterine gas gangrene, particularly that caused by *C. sordellii*, has historically been associated with illegal or self-induced abortions, but in modern practice has also been reported after spontaneous abortion, normal vaginal delivery, and cesarean section [7]. Fatal postpartum *C. sordellii* infections in previously healthy young women are characterized by a distinctive clinical presentation, including minimal or absent fever, lack of purulent discharge, refractory hypotension, massive peripheral edema and pleural effusion, hemoconcentration, and marked leukocytosis [8]. These cases progress rapidly and are frequently fatal. Similarly, gas gangrene and necrotizing endometritis caused by *C. perfringens *have been reported with 30-day mortality rates ranging from 27% to 44% [9]. Infections due to clostridial species may arise from ascending or iatrogenic routes, and are challenging to diagnose because of their fulminant course, nonspecific symptoms, and the low rate of positive blood cultures [5]. Recommended treatment consists of prompt surgical debridement combined with antimicrobial therapy, typically penicillin G in combination with clindamycin, or alternatively agents such as MEPM or MNZ [10]. Thus, although some *Clostridium* species are associated with gynecological infections, to our knowledge, there have been no reports of such infections caused by *C. clostridioforme*.

In our case, multiple bacteria were isolated from the pyometra, and the isolated bacterial profile changed during antibiotic therapy. Initially, *P. mirabilis* and *E. faecalis* were isolated from the uterine cavity, both susceptible to MEPM. However, after systemic administration of MEPM, subsequent cultures revealed the emergence of* C. clostridioforme*. Although *C. clostridioforme* was not detected in the initial cultures, given that detection of anaerobes is more challenging than detection of aerobes, we speculate that the original infection was polymicrobial, involving at least these three organisms; nonetheless, only *C. clostridioforme* was recovered after MEPM treatment. This pattern suggests that antimicrobial penetration into the infected uterine tissue was insufficient to eradicate this anaerobe.

The differential efficacy observed in this case can be explained by the pharmacokinetic limitations of systemic carbapenem administration in uterine infections. Gall et al. demonstrated that following intravenous administration of 500 mg MEPM, endometrial tissue concentrations reached only 2.3 μg/g at peak levels [11]. While these concentrations may be adequate for susceptible aerobic pathogens like *P. mirabilis* and *E. faecalis*, they may prove insufficient for more resistant anaerobic organisms, particularly in the presence of purulent material that can further impair drug penetration. This finding is consistent with previous studies showing superior tissue concentrations with local versus systemic antibiotic administration. Braun et al. reported significantly higher antimicrobial concentrations in uterine tissue layers following intrauterine instillation compared to systemic administration, with the disparity being most pronounced in deeper tissue layers where anaerobic bacteria typically proliferate [12]. The authors concluded that "systemic treatment resulted in lower tissue concentrations compared to intrauterine treatment, particularly for anaerobic bacterial coverage." Furthermore, they emphasize that combined systemic and local antibiotic therapy is likely optimal for treating mixed infections in restricted spaces like the uterine cavity, considering the superior tissue penetration achieved through intrauterine instillation [12].

In addition, antibiotic resistance in *C. clostridioforme*, including resistance to carbapenems, is a significant concern. A comprehensive genomic analysis of *C. clostridioforme *strains by Dehoux et al. revealed the presence of multiple β-lactam resistance determinants, such as class A, C, and D β-lactamases and metallo-β-lactamases [3]. Therefore, due to the potential for antibiotic resistance and the challenges of achieving adequate endometrial penetration, treatment of pyometra caused by *C. clostridioforme* should involve a combination of systemic and local antibiotic administration, in conjunction with uterine cavity lavage.

While the isolate demonstrated susceptibility to clindamycin, MNZ was chosen as the primary treatment for several reasons. MNZ is a well-established agent with potent activity against anaerobic bacteria, including *C. clostridioforme*, and it possesses excellent tissue penetration, particularly in abscesses and pelvic infections [13]. In addition, MNZ has been reported to achieve superior clinical and bacteriological cure rates in pyometra and gynecological anaerobic infections compared to other treatments, as evidenced by veterinary and clinical studies [13]. Although neurological adverse effects such as peripheral neuropathy are recognized risks with MNZ [14], especially with prolonged use or in older adults, the treatment duration in this case was limited, reducing this risk substantially. Furthermore, clindamycin use carries potential risks, including *Clostridioides difficile* infection and emerging resistance among anaerobes. Given the clinical urgency, susceptibility profile, and favorable safety when used appropriately, MNZ was considered the most effective and reliable option for this case. Nonetheless, we acknowledge the need for careful monitoring of neurological symptoms during therapy, particularly in the elderly. Future cases may warrant considering clindamycin as an alternative based on individual patient risk factors.

## Conclusions

This report describes a rare case of pyometra caused by *C. clostridioforme*. This case highlights the importance of integrating pharmacokinetic considerations with microbial etiology in the management of complex uterine infections. In similar cases, a treatment strategy combining systemic antibiotics with local measures such as endocervical lavage may be beneficial, but it does not guarantee a cure. Additional studies are needed to elucidate the pathophysiology underlying *C. clostridioforme*-associated pyometra and to establish optimal therapeutic approaches.
